# Supplementary material for: Serotonin drives aggression and social behaviors of laboratory male mice in a semi-natural environment
Source: Front Behav Neurosci. 2024 Sep 18;18:1450540. doi: 10.3389/fnbeh.2024.1450540 (PMC11446219; doi:10.3389/fnbeh.2024.1450540)

SUPPLEMENTARY Figure 1. Behaviour occurrences during dark and light phases separately. Wilcoxon-Mann-Whitney test, * p < 0.05 between the genotypes.


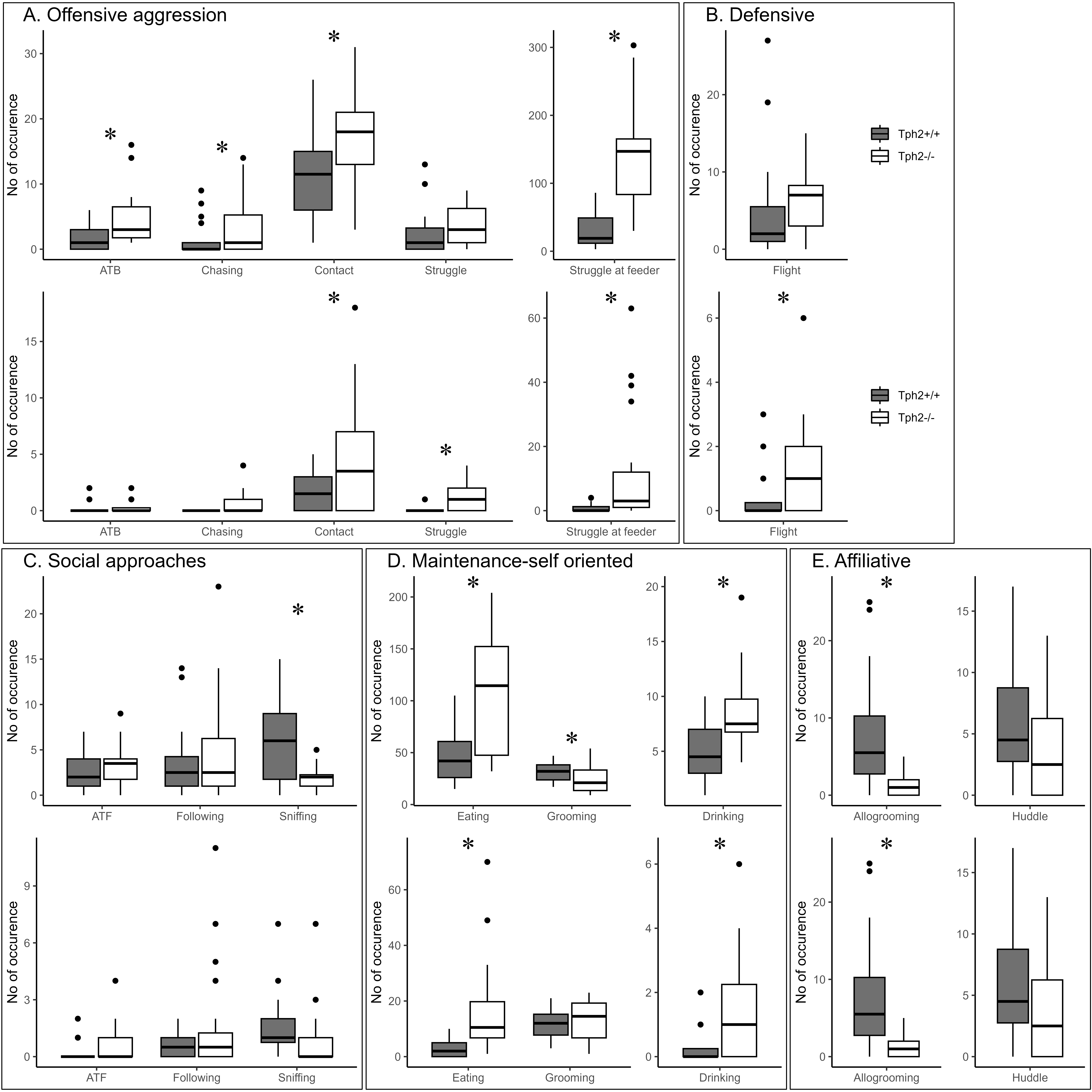


SUPPLEMENTARY Table 1. Correlation table of VBS behaviours of *Tph2*^+/+^ mice

| **Correlation Matrix** | | | | | | | | | | | | | | | | | | | | | | | | | | | | | | | | | | | | | |  |  |  |
| --- | --- | --- | --- | --- | --- | --- | --- | --- | --- | --- | --- | --- | --- | --- | --- | --- | --- | --- | --- | --- | --- | --- | --- | --- | --- | --- | --- | --- | --- | --- | --- | --- | --- | --- | --- | --- | --- | --- | --- | --- |
|  | | chasing | | | | contact | | | struggle | | | | SAF | atb | | | | eat | | sniff | | | groom | | | drink | | | allogroom | | | | | huddle | | | |  |  |  |
| chasing |  | | — |  | **0.45** | | |  | | **0.6** |  | 0.268 | | |  | 0.173 |  | | 0.238 | |  | 0.5 | |  | -0.216 | |  | 0.130 | |  | 0.195 |  | | | **-0.676** | |  | | |  |
| contact |  | |  |  | — | |  | | | **0.5** |  | 0.205 | | |  | **0.630** |  | | 0.173 | |  | 0.7 | |  | 0.051 | |  | 0.199 | |  | -0.003 |  | | | -0.426 | |  | | |  |
| struggle |  | |  |  |  | |  | | | — |  | -0.102 | | |  | 0.386 |  | | -0.319 | |  | 0.7 | |  | 0.254 | |  | -0.130 | |  | 0.183 |  | | | **-0.713** | |  | | |  |
| SAF |  | |  |  |  | |  | | |  |  | — | | |  | -0.012 |  | | **0.813** | |  | -0.2 | |  | **-0.573** | |  | **0.476** | |  | **-0.653** |  | | | -0.169 | |  | | |  |
| atb |  | |  |  |  | |  | | |  |  |  | | |  | — |  | | -0.048 | |  | **0.61** | |  | -0.114 | |  | -0.216 | |  | 0.117 |  | | | 0.106 | |  | | |  |
| eat |  | |  |  |  | |  | | |  |  |  | | |  |  |  | | — | |  | -0.28 | |  | **-0.759** | |  | **0.623** | |  | **-0.629** |  | | | -0.033 | |  | | |  |
| sniff |  | |  |  |  | |  | | |  |  |  | | |  |  |  | |  | |  | — | |  | 0.156 | |  | -0.052 | |  | **0.451** |  | | | **-0.441** | |  | | |  |
| groom |  | |  |  |  | |  | | |  |  |  | | |  |  |  | |  | |  |  | |  | — | |  | -0.435 | |  | 0.415 | |  | | | -0.189 | | |  | |
| drink |  | |  |  |  | |  | | |  |  |  | | |  |  |  | |  | |  |  | |  |  | |  | — | |  | -0.346 |  | | | -0.274 | |  | | |  |
| allogroom |  | |  |  |  | |  | | |  |  |  | | |  |  |  | |  | |  |  | |  |  | |  |  | |  | — |  | | | 0.040 | |  | | |  |
| huddle |  | |  |  |  | |  | | |  |  |  | | |  |  |  | |  | |  |  | |  |  | |  |  | |  |  |  | | | — | |  | | |  |
| Note.  Spearman correlation; **Bold** <0.05 {jamovi} | | | | | | | | | | | | | | | | | | | | | | | | | | | | | | | | | | | | | |  |  |  |

SUPPLEMENTARY Table 2. Correlation table of VBS behaviours of *Tph2*^-/-^ - mice

| **Correlation Matrix** | | | | | | | | | | | | | | | | | | | | | | | | | | | | | | | | | |
| --- | --- | --- | --- | --- | --- | --- | --- | --- | --- | --- | --- | --- | --- | --- | --- | --- | --- | --- | --- | --- | --- | --- | --- | --- | --- | --- | --- | --- | --- | --- | --- | --- | --- |
|  | chasing | | | contact | | struggle | | SAF | | atb | | | eat | | | sniff | | groom | | | | drink | | | allogroom | | | | huddle | | |  |  |
| chasing |  | — |  | -0.189 |  | 0.395 |  | -0.110 |  | | 0.424 |  | | -0.231 |  | | 0.273 | |  | -0.122 |  | | 0.124 |  | | 0.415 |  | | | 0.240 |  | |  |
| contact |  |  |  | — |  | 0.246 |  | **0.586** |  | | **-0.542** |  | | **0.547** |  | | -0.364 | |  | 0.036 |  | | 0.151 |  | | -0.084 |  | | | **-0.633** |  | |  |
| struggling |  |  |  |  |  | — |  | 0.387 |  | | 0.056 |  | | 0.259 |  | | 0.229 | |  | 0.118 |  | | 0.255 |  | | 0.106 |  | | | -0.061 |  | |  |
| SAF |  |  |  |  |  |  |  | — |  | | **-0.667** |  | | **0.936** |  | | -0.135 | |  | 0.286 |  | | 0.347 |  | | -0.354 |  | | | **-0.735** |  | |  |
| atb |  |  |  |  |  |  |  |  |  | | — |  | | **-0.782** |  | | 0.361 | |  | **-0.503** |  | | -0.163 |  | | **0.613** |  | | | **0.771** |  | |  |
| eating |  |  |  |  |  |  |  |  |  | |  |  | | — |  | | -0.134 | |  | 0.351 |  | | 0.254 |  | | **-0.483** |  | | | **-0.723** |  | |  |
| sniffing |  |  |  |  |  |  |  |  |  | |  |  | |  |  | | — | |  | -0.001 |  | | -0.157 |  | | 0.092 |  | | | 0.292 |  | |  |
| grooming |  |  |  |  |  |  |  |  |  | |  |  | |  |  | |  | |  | — |  | | 0.246 |  | | -0.067 |  | | | -0.429 |  | |  |
| drinking |  |  |  |  |  |  |  |  |  | |  |  | |  |  | |  | |  |  |  | | — |  | | 0.037 |  | | | -0.204 |  | |  |
| allogrooming |  |  |  |  |  |  |  |  |  | |  |  | |  |  | |  | |  |  |  | |  |  | | — |  | | | 0.214 |  | |  |
| huddle |  |  |  |  |  |  |  |  |  | |  |  | |  |  | |  | |  |  |  | |  |  | |  |  | | | — |  | |  |
| Note.  Spearman correlation; **Bold** <0.05 {jamovi) | | | | | | | | | | | | | | | | | | | | | | | | | | | |  |  |  |  |  |  |

With some exceptions, *Tph2*^-/-^ mice presented equivalent relationships between variables than *Tph2*^+/+^ mice (correlations p>0.05). In both groups occurrence of “eating” positively correlates with the occurrence of “struggle at feeder” (Tab. S1, S2) and “struggle at feeder” (and “eating”) negatively correlated with affiliative behaviours such as “huddling” in *Tph2*^-/-^ mice and “allogrooming” and also “grooming” in *Tph2*^+/+^ group. Only in the *Tph2*^+/+^ group “huddling” negatively correlated with “chasing”, “struggle” and “sniffing”. In *Tph2*^+/+^ groups, the occurrence of a “contact” was positively associated with the occurrence of “approach to back” (ATB), “chasing” and “struggle” (Table S1, S2) while surprisingly, in *Tph2*^-/-^mice, “contact” was positively related to “struggle at feeder” (and “eating”) but negatively to “ATB” (and “huddle”) (Tab. S2). Only in the *Tph2*^+/+^ group “chasing”, “contact” and “struggle” covariated positively. In the *Tph2*^+/+^ mice “sniffing” positively correlated with “allogrooming” and “ATB” and negatively with “huddling”. In *Tph2*^-/-^ group “ATB” was the behaviour which correlated with most of the other scored behaviours. It was positively related to “huddling” and “allogrooming” but negatively to the occurrence of “struggle at feeder”, “grooming” and “eating”. Finally, in the *Tph2*^-/-^ group, “allogrooming” negatively correlated with “eating”.

SUPPLEMENTARY Figure 3. Glicko rating of all groups.

Top line: *Tph2*^+/+^,

Bottom line: *Tph2*^-/-^

In the *Tph2*^+/+^ groups the higher ranked individual (highest final Glicko rating at the end of a VBS stay) already emerged as the most dominant animal after a median of 68.75% of all agonistic interactions (mean: 64.46% ± SD: 34.08%; group scores: 10.19%, 100%, 44.53%, 98.84%, 68.75%). In the *Tph2*^-/-^groups, the dominant males at the end of a VBS emerged as the dominant individual of its group after a median of 80.08% of all agonistic interactions (mean: 80.3 ± SD: 12.43%; group scores: 85.04%, 73.41%, 62.95% 80.08%, 100%).

SUPPLEMENTARY Table 3.

|  | ***Tph2*^+/+^** | |  |  | ***Tph2^-/-^*** | |
| --- | --- | --- | --- | --- | --- | --- |
|  | **Group** | **Power of dominant** |  |  | **Group** | **Power of dominant** |
|  | 1 | 0.67 |  |  | 6 | 0.36 |
|  | 2 | 0.24 |  |  | 7 | 0.08 |
|  | 3 | 0.34 |  |  | 8 | 0.29 |
|  | 4 | 0.35 |  |  | 9 | 0.10 |
|  | 5 | 0.52 |  |  | 10 | 0.67 |
| **Mean** |  | **0.42** |  | **Mean** |  | **0.30** |
| **Variance** |  | **0.03** |  | **Variance** |  | **0.06** |

The power of the dominant male was evaluated as a ratio (relative proportion) of power, defined as the difference in Glicko rating the dominant male is imposing on the first subordinate male (second highest Glicko rating score) compared to the power projected from the dominant male to the most subordinate animal (lowest Glicko rating score). A high value represents a more strongly despotic dominant male imposing relatively similar amounts of power towards all other animals.

SUPPLEMENTARY Figure 4. Social recognition in the three-chamber test. Total duration of interaction with the enclosed familiar mouse and the enclosed unfamiliar mouse during minutes 1-5 (A) and 6-10 (B) of test in seconds.


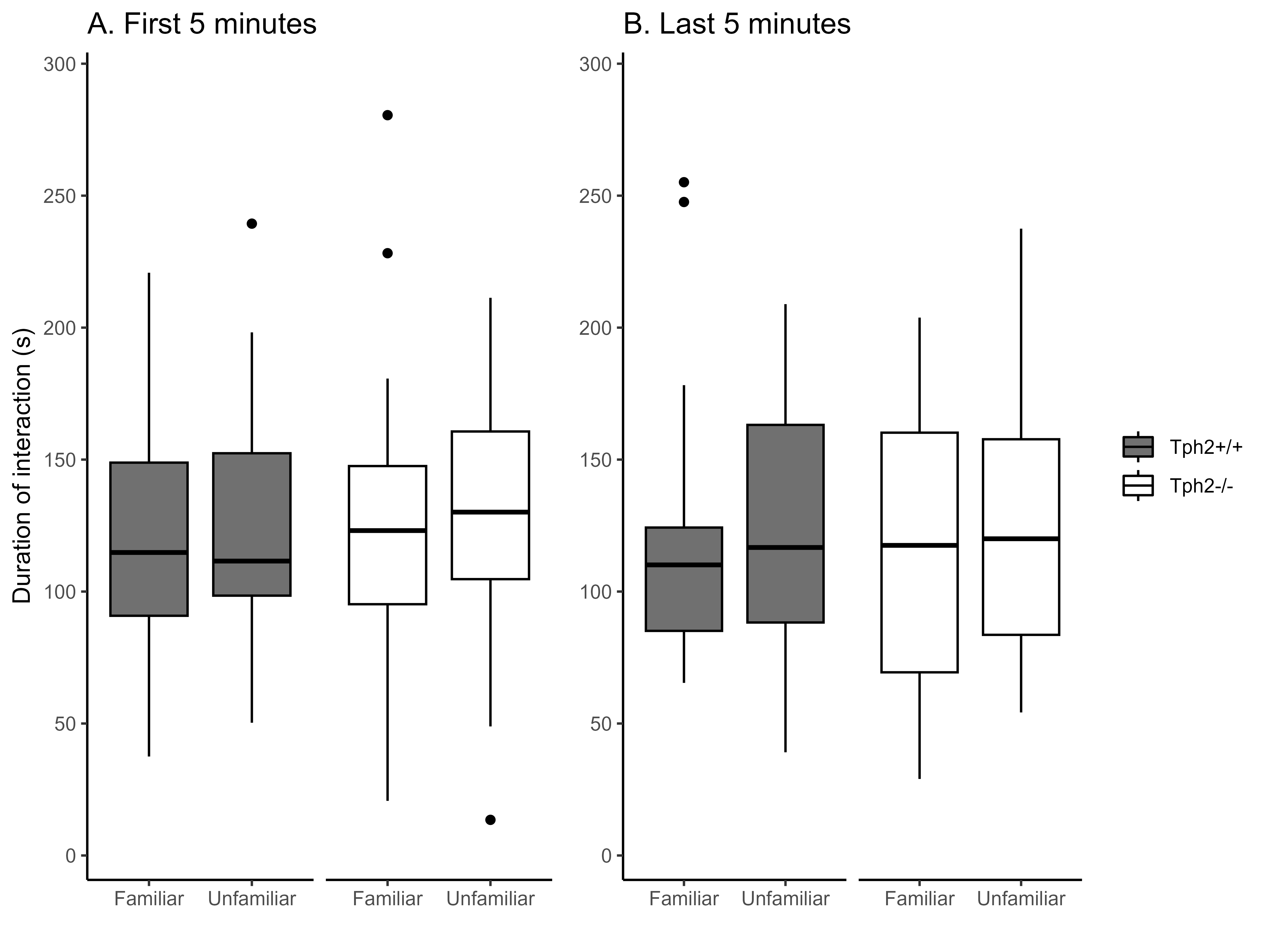

Supplement: Supplementary file 1 [file Data_Sheet_1.docx]
